# Dr. H. C. Proctor

**Published:** 1910-05-28

**Authors:** 


We have to record the death of Dr. H. C. Proctor. He studied at Leeds and at the Middlesex Hospital, taking his diploma in 1876. During the Zulu War he went out as a civil surgeon to South Africa, with a practice first at Harrismith, and then at Ladysmith. He graduated M.D. at Brussels in 1901, and returned to England a few years later. His death occurred at Neasden, near London.

